# Deconstructing Participant Behaviors in Virtual Reality Simulation: Ethnographic Analysis

**DOI:** 10.2196/65886

**Published:** 2025-10-27

**Authors:** Daniel Loeb, Jamie Shoemaker, Kelly Ely, Matthew Zackoff

**Affiliations:** 1 Division of Critical Care Medicine, Cincinnati Children's Hospital Medical Center, 3333 Burnett Avenue, Cincinnati, OH, 45229, United States, 1 216-299-0004; 2 Department of Pediatrics, University of Cincinnati College of Medicine, Cincinnati, OH, United States; 3 Center for Simulation and Research, Cincinnati Children’s Hospital Medical Center, Cincinnati, OH, United States

**Keywords:** simulation, virtual reality, video review, resuscitation, CPR, immersion, VR, ethnographic analysis, tool, respiratory distress, pneumonia, sepsis, children, scenarios, ethnography, VR-based simulation, training, training method, development, cardiopulmonary resuscitation

## Abstract

**Background:**

Virtual reality (VR)–based simulation is an increasingly popular tool for simulation-based medical education, immersing participants in a realistic, 3D world where health care professionals can observe nuanced examination findings, such as subtle indicators of respiratory distress and skin perfusion. However, it remains unknown how the VR environment affects participant behavior and attention.

**Objective:**

This study aimed to describe clinician attention and decision-making behaviors during interprofessional pediatric resuscitation simulations performed in VR. We used video-based focused ethnography to describe how participant attention and behavior are altered in the VR environment and reflect how these changes may affect the educational profile of VR simulation.

**Methods:**

The research team analyzed scenarios with the question, “How does a completely virtual reality environment alter participant attention and behavior, and how might these changes impact educational goals?” Video-based focused ethnography consisting of data collection, analysis, and pattern explanation was conducted by experts in critical care, resuscitation, simulation, and medical education until data saturation was achieved.

**Results:**

Fifteen interprofessional VR simulation sessions featuring the same scenario—a child with pneumonia and sepsis—were evaluated. Three major themes emerged: Source of Truth, Cognitive Focus, and Fidelity Breakers. Source of Truth explores how participants gather and synthesize information in a VR environment. Participants used the patient’s physical examination over ancillary data sources, such as the cardiorespiratory monitor, returning to the monitor when the examination did not align with expectations. Cognitive Focus describes the interplay between thinking, communicating, and doing during a VR simulation. The VR setting imposed unique cognitive demands, requiring participants to process information from multiple sources, make rapid decisions, and execute tasks during the scenario. Participants experienced increased task burden when virtual tasks did not mirror real-world procedures, leading to delays and fixation on certain actions. Fidelity Breakers reflects how technical and environmental factors disrupted focus and hindered learning. Navigational challenges, such as unintended teleportation and difficulties interacting with the virtual patient and equipment, disrupted participant immersion. These challenges underscore the current limitations of VR in reproducing the tactile and procedural aspects of real clinical care.

**Conclusions:**

Participants’ focus on the physical examination findings in VR, as opposed to the cardiorespiratory monitor, potentially indicates simulation of an identical, more patient examination-centered approach to clinical data gathering. In addition, the multiple data sources allowed for participant cognitive load and task burden that may better mirror real-life clinical care. However, technical features that required straying from real-world task completion, as well as other navigational and interactional challenges in VR, led to breaks in fidelity and shifted focus away from the learning objectives. These findings underscore the need for continued research on how simulation modality, fidelity, and technical challenges may influence participant attention and behavior, to allow thoughtful alignment between desired learning objectives and mode of training.

## Introduction

Simulation-based medical education (SBME) emerged as a way to practice skills without placing patients at risk [1]. Simulation has been shaped by the tools and technology available and has primarily consisted of ever-evolving computerized manikins designed to represent a patient. Computerized manikins have been well aligned with training toward and evaluation of specific goals and behaviors, such as practicing high-quality cardiopulmonary resuscitation [2], enhancing team dynamics [3], and establishing procedural competency [4]. Recently, technological advancements have allowed SBME to move beyond computerized manikins. Digital simulation environments can allow for a simulated patient to exist in 3D space as a virtual patient. These new approaches to simulation change the way participants perceive and interact with the patient during training scenarios [5-10].

Institutions have begun to explore the use of several digital-based simulation modalities, including augmented reality (AR) and virtual reality (VR)–based simulation. AR simulation places a 3D virtual patient in an actual physical space [11]. This virtual patient can either exist alone or as a superimposed image anchored onto a physical manikin [12]. AR passthrough technology obviates the need to replicate a physical environment in a virtual space, since the simulation occurs in the physical world. However, the technology needed to allow a seamless passthrough AR experience remains limited [13].

Immersive VR places participants in a fully digital 3D environment. This environment can be a digital twin of the actual clinical space used by the participants [12,14] or a completely novel training environment [8]. Achieving high-fidelity physical interactions and closely matching real-life clinical skills in VR remains challenging [15].

SBME is built upon the foundational presumption that the training environment, and the associated degree of realism and fidelity, impacts learning through the quantity and complexity of cognitive tasks required (ie, cognitive load theory) [16], as well as through allowing new knowledge construction through experiences and interactions (ie, constructivism) [17]. Augmented and VR, which significantly change the environment where participants engage, learn, and grow, could influence the attention (ie, directed cognitive engagement) and behaviors (ie, observable participant actions) exhibited by participants. In addition, while some of this influence may be purposeful (ie, realistic presentation of clinical findings), it is unknown whether there are additional influences that may be inherent to the training modality and independent of the clinical context and learning objectives. While these new and innovative tools present opportunities for training and assessment, an understanding of their influence on trainee attention and behavior is necessary to optimize our ability to use them to achieve ideal educational outcomes.

Our previous findings found that participant behaviors differed substantially between an AR-enhanced and a manikin-based simulation of an identical clinical scenario. The modality influenced the observed communication dynamics, participant behaviors, and fidelity breakpoints that influenced participant attention [5]. However, a similar description of attention and behavior in a VR clinical simulation currently does not exist. As these digital training modalities are becoming increasingly accessible, addressing this gap is crucial for supporting educators in developing and implementing precision training that is potentially more impactful through thoughtful alignment between learning objectives and chosen training modality. This study aimed to identify and categorize provider attention and behavior during VR SBME to aid in establishing which educational objectives are best addressed through this novel simulation modality.

## Methods

### Theoretical Framework

This study is anchored in the theoretical construct that the inherent characteristics of a simulation modality may influence participant attention and behavior. In addition, understanding that influence is key to ensuring that specific learning objectives are feasible to accomplish with the use of a given simulation modality. This aligns with the educational theory of constructivism, which posits that learners actively build knowledge by engaging in “hands-on” experiences, experimentation, and reflection within authentic contexts [17]. In a VR simulation, these authentic contexts are approximated by realistic clinical environments that include realistic digital patients and equipment to encourage exploration, role adoption, and collaborative decision-making. However, the same immersive features that make VR engaging (eg, richly detailed visuals, interactivity, and collaboration) may also impose additional cognitive demands (eg, technical work-arounds or deviations from reality) that could influence the construction of knowledge in unintended ways. The impact of those cognitive demands can best be understood through the lens of cognitive load theory, which highlights the limited capacity of working memory and its subsequent impact on knowledge or skill acquisition [16]. With VR simulations, expanded data sources in the form of virtual patients, equipment, environments, or other assets will influence the cognitive load burden on participants and potentially influence their behavior and subsequent learning. At the same time, novel controls and interface complexity may inadvertently add additional cognitive load, drawing learners’ mental resources away from essential clinical tasks and therefore potentially diminishing their bandwidth for the desired learning objectives. The integration of constructivism and cognitive load theory allows for a theoretical framework that aims to capture how participants effectively manage mental resources while constructing new knowledge through immersion, as well as identify when extraneous load hinders that process. This blended theoretical lens guided our data analysis and interpretation of observed learner behaviors in VR-based pediatric resuscitation simulations.

### Study Design

We used video-based focused ethnography [18,19] to study a cohort of video-recorded VR simulations. Focused ethnography is a methodological adaptation of traditional ethnography, offering a targeted and time-efficient approach to studying specific phenomena within specific contexts. This research strategy prioritizes depth of understanding over breadth, using concentrated data collection and a clear research question to describe particular social, cultural, or organizational processes. For this study, the goal was to understand how VR-based simulation affects participant attention and behavior. With this goal in mind, the research team was primed with the initial research question [19]: “How does a virtual reality simulation influence participant attention and behavior, and how might these changes impact educational goals?”

During this focused exploration, the team first identified and classified the data, then described and analyzed the specific behaviors, and finally moved to pattern explanation [18,19]. This study was approved by the Institutional Review Board at Cincinnati Children’s Hospital Medical Center. It was funded, in part, by the Laerdal Foundation.

### Data Corpus

Our research team, composed of an interdisciplinary group with combined expertise in critical care (DL, KE, and MZ), resuscitation (DL and MZ), simulation (DL, KE, and JS), and medical education (DL, JS, and MZ), reviewed a series of VR simulation recordings captured during a validation study of a specific VR curriculum [20]. The simulation took place in a virtual environment that mirrored a typical high-acuity room, as might exist in an emergency department or intensive care unit. Participants in the simulation included pediatric critical care and emergency medicine attendings and fellows, and pediatric intensive care unit and emergency department nurses. Each physician-nurse dyad had no previous exposure to the scenario and completed it only once.

**Figure 1.**
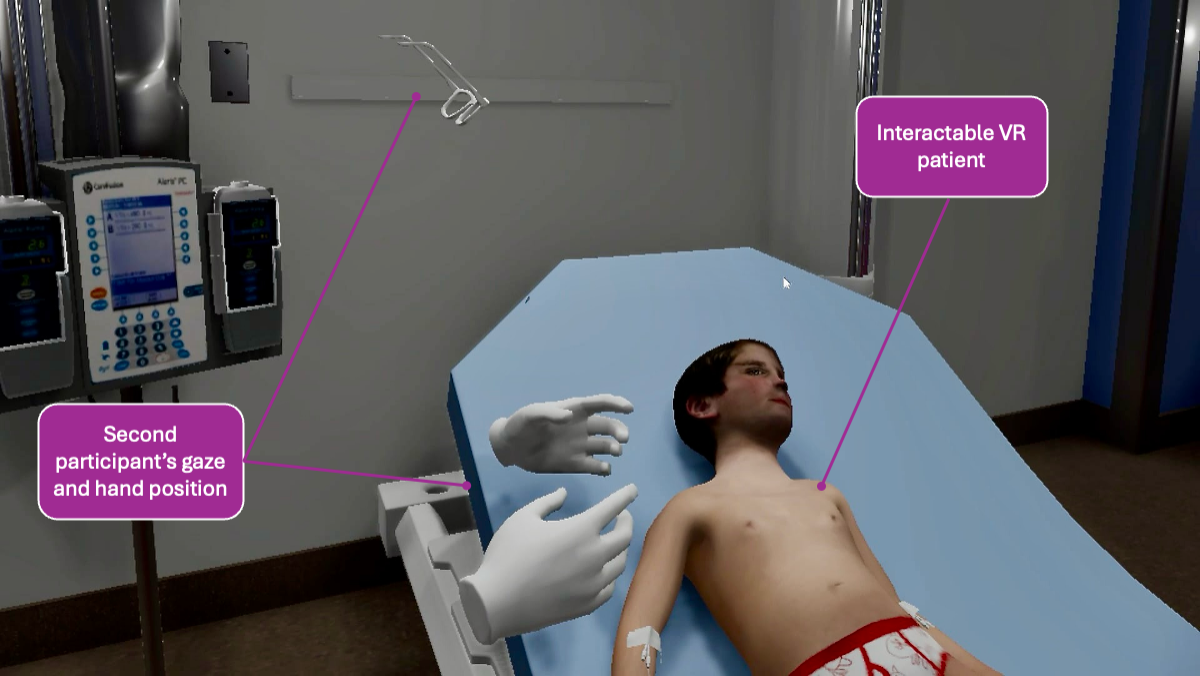
First-person perspective of the virtual reality (VR) experience.

The virtual patient was located on a hospital bed within a room that included a vital sign monitor, an intravenous pole with fluids, an oxygen delivery device, a stethoscope, a thermometer, a syringe for drawing blood samples, and a large fluid-filled syringe for triggering administration of a fluid bolus. A representative first-person perspective of the VR environment can be found in Figure 1. The virtual patient was an 8-year-old male with pneumonia and sepsis. The rationale for this scenario was that pneumonia frequently progresses to sepsis, and key stakeholders regarded this as a realistic scenario for clinical assessment and management training. The clinical status of the virtual patient changed throughout the simulation, conveying alterations in mental status (ranging from conversant to altered), perfusion (mottled skin that progressed to poor perfusion and cyanosis), and respiratory status (superimposed retractions and tachypnea).

The scenario was coded in Unity (Unity Technologies) and accessed via an Oculus Rift headset connected to a VR-capable laptop, which allowed 2 participants within the same simulation to move freely and interact with the scenario and each other, including communicating regarding shared findings in real time. The purpose of the simulation was to assess the participants’ ability to recognize, describe, and begin to treat a patient with developing shock. A detailed description of this VR simulation was previously described by Zackoff et al [20]. Participants received a 5-minute orientation to navigation in the VR environment and the functionality of the virtual patient and equipment from a trained VR simulation facilitator. They then received the case description and were instructed to begin their assessment and management of the patient as they would in real life. The VR simulation facilitator was present in the VR environment for the orientation and then exited the VR but remained present in real life to address questions and provide feedback throughout the remainder of the simulation session.

First-person audiovisual data recorded from the perspective of both users (a nurse and a physician) were stored in a password-protected, encrypted server, annotated via Vimeo (Vimeo, Inc), and coded in Microsoft Excel. Multiple audiovisual feeds, capturing the perspective of both participants within the virtual environment, allowed for data triangulation [21].

### Research Team

Considering reflexivity [22] and the desire for analytic triangulation [21], the research team was composed of a heterogeneous group of experts in resuscitation team leadership and medical education (DL), resuscitation bedside nursing (KE), and SBME (JS). DL is a practicing pediatric critical care physician as well as a simulation educator. KE is a simulation educator and former critical care nurse. JS is a simulation educator and a former pediatric emergency department nurse. The data analysis was also overseen by MZ, a pediatric critical care physician and education scientist with expertise in designing, implementing, and evaluating SBME using innovative modalities such as VR and AR. MZ served as a senior consultant, providing guidance and expertise at scheduled intervals and during critical decision points where consensus within the research team was required.

### Focused Research Question

The research team reviewed the data, primed with the following generative question [23], “How does a virtual reality simulation influence participant attention and behavior, and how might these influences impact achievable educational goals?”

Using a focused ethnographic approach is particularly suited for foundational research in which little is known about how subjects interact with a new environment (or, more specifically, a technology that places subjects in a new environment). Rather than measure changes against a specific baseline or control group, a focused ethnographic approach provides foundational descriptions of behaviors [18]. Instead of benchmarking against manikin-based or live simulations, our goal was to capture and interpret emergent behaviors, areas of challenge, and decision-making processes that may be uniquely influenced by VR-based training.

### Phased Qualitative Analysis of Participant Attention and Behavior

To investigate participant attention and behavior within a virtual environment, we conducted a 3-phase analysis of VR simulations [18,24]. We followed a focused ethnographic strategy [18,19,24] incorporating inductive coding techniques [25]. This approach was chosen to (1) provide in-depth, context-specific insights into participant behavior in VR and (2) allow unanticipated themes to emerge from the data rather than imposing pre-existing hypotheses.

#### Phase 1: Identification and Classification

The initial phase involved exposing the research team to the VR scenarios. All participants were oriented to the technology, simulation, and virtual environment. The team then watched an initial group of scenarios to further familiarize themselves with the simulation and VR environment. For each study video, each researcher took independent field notes [26]—timestamped observations documenting participant statements, team dynamics, and points of interest [18]. These notes were stored, compiled, and subsequently treated as data and shared during collective analysis sessions.

The units of analysis for these notes were discrete, timestamped episodes of participant behavior. Team members independently categorized these notes into two broad types: (1) verbal events: any distinct verbalization by a participant (eg, “I’m going to give a bolus,” or “He looks cyanotic”) and (2) Interaction events: any observed action in the VR environment (eg, checking capillary refill, reaching for oxygen supplies, and teleporting to a different location). This unit of analysis helped capture how participants directed attention, interpreted clinical cues, and engaged with the virtual environment.

During the collective analysis sessions, the research team met together and used triangulation techniques to reconcile discrepancies in individual coding and ultimately reach consensus during the development of a comprehensive codebook. The team compared individual code lists, resolved discrepancies through discussion, and compiled a unified preliminary codebook. During this stage, our theoretical framework served as a sensitizing concept, alerting us to phenomena related to the active construction of knowledge and influence of cognitive load (eg, repeated attempts to use virtual equipment, indicating extraneous load on top of the active construction of clinical assessment knowledge).

This codebook served as a standardized tool for subsequent research phases (Phase 2: Description and Analysis and Phase 3: Pattern Explanation). Following the consolidation of the codebook, the research team independently applied it to each VR simulation session video recording. After coding each simulation session, the team reconvened to discuss and refine the codebook further as needed. This iterative process continued until data saturation [27] was achieved.

#### Phase 2: Description and Analysis

After achieving data saturation, the research team transitioned to the second phase—description and analysis. A rigorous review of the developed codebook was conducted to identify recurring patterns in participant attention and behavior across the reviewed simulated scenarios, guided by the focused research question.

The recurring findings were subsequently organized into distinct clusters to inform themes, providing a comprehensive overview of participant engagement during the VR simulations. To ensure the validity and consistency of these themes, an independent researcher (MZ) again triangulated the data.

#### Phase 3: Pattern Explanation

The final phase used the established themes to facilitate a descriptive analysis of the potential ramifications of using VR as an educational tool. By exploring the patterns of attention and behavior exhibited during VR-based simulations, we identified unique advantages and limitations associated with this modality. We reflected on how these insights may aid educators in determining which educational objectives are best aligned with VR training.

### Ensuring Code Validity

We used multiple strategies to enhance the credibility and dependability of our findings. First, we used analyst triangulation [21,28] by involving researchers with diverse backgrounds in critical care, nursing, and simulation education. Each analyst independently reviewed and coded selected video segments, then convened to discuss and reconcile any discrepancies. This iterative group process helped refine the codebook, ensuring that multiple perspectives were integrated and minimizing the influence of individual bias. We also maintained reflexive memos [22] to document personal assumptions or theoretical leanings; these memos were revisited throughout data analysis to encourage ongoing reflection on how our backgrounds might shape interpretations.

In addition, we created an audit trail [29] that outlined key decisions made during coding and theme development. This trail included records of coding updates, consensus discussions, and rationale for code merging or splitting, providing a transparent account of how the analysis evolved. By combining triangulation, reflexivity, and rigorous documentation, we sought to strengthen the trustworthiness of our findings and facilitate replicability of the study’s analytic approach.

### Ethical Considerations

The primary study and this secondary analysis received approval from the Cincinnati Children’s Hospital Institutional Review Board, which granted a waiver of documentation of informed consent in accordance with 45 CFR 46.116(d). This waiver was permissible because the research posed no more than minimal risk to participants, did not compromise their rights and welfare, could not be feasibly conducted without the waiver, and would provide subjects with additional relevant information after participation, when appropriate. Given its educational focus and the absence of risk to participants, the study met these criteria. Participation in the simulations was voluntary, with no compensation provided. All data on participant performance were stored securely on a password-protected server.

## Results

### Overview

The ethnographic analysis was conducted on the recordings of 15 interprofessional teams of 2 users (a nurse and a physician) who completed the VR simulation. The simulations were recorded from both the perspective of the nurse and the team lead. This analysis revealed 3 major themes and associated subthemes that offer insights into the interplay between simulation modality and participant attention and behavior (Table 1).

**Table 1.** Ethnographic analysis results.

Themes, subthemes, and their examples	Illustrative descriptors and quotes
Source of Truth	
Initial reliance on virtual cues	
Observing movements	The nurse and team lead observe the virtual patient swatting at the team with his hands and struggling to breathe. The nurse states, “Pending a [blood gas to confirm], we are probably looking at assisting ventilation one way or another.” [N11]
Mental status	“Quick cardiopulmonary assessment–His mental status is down; his airway is open. I need to take a listen to him, but I can see him breathing, cap refill is about 3‐4 seconds, he looks like crap… I am thinking shock, likely sepsis.” [P8] “Timmy? HELLOO?” Meanwhile, the team lead Interacts with the virtual patient’s foot and then performs a sternal rub. [P6]
Linking physical examination to clinical progression	After evaluating the patient, the team lead states, “Airway seems to be ok. He seems to be a little more alert.” Asks the virtual patient, “How you doing buddy?” The team lead taps on that patient’s chest, and while sounding stressed, says, “He isn’t responding.” As the virtual patient starts to respond, the team lead sounds calmer. [P13] Team lead says the virtual patient has bounding “femoral” and “jugular” pulses as he palpates them sequentially. [P2]
Dynamic reliance based on response	
Increasing reliance on the cardiopulmonary monitor when the examination does not change as expected	“[After receiving fluid, the] blood pressure is still a little low, so that may be the cause of his tachycardia, but he also has fever, so…(provider does not finish sentence).” [P5] Midway through the simulation, the team lead notes that the patient still has poor perfusion and is still working hard to breathe. He recognizes that about half of the fluid bolus is in, yet the patient has not improved. He then turns to the monitor, remarking on the tachycardia and hypoxemia. He asks for vancomycin to be added while staring at the monitor. [P10]
Cognitive Focus	
Task burden and scenario complexity	
Synthesizing large amounts of information quickly	Team lead listens to the lungs, looks at the monitor, and states*, “*He looks kind of like ass... why is his face purple?” [P3] After giving a fluid bolus, the team lead reports, “His heart rate is trending down nicely.” [P5] The nurse responds, “His perfusion in his hands is 3‐4 seconds.” [N5] The team lead leans over the virtual patient and watches the cap refill animation.
Transitioning from interpretation to action	“He looks sick, he is a little cyanotic...can you put oxygen on him?” [P13] The team lead requests, “septic workup stuff.” Quickly asks for several interventions, and then asks the nurse for suggestions. [P8]
Cognitive demands as an unexpected consequence of simulation modality	
Completing examination in VR^a^	The team lead reaches for the stethoscope on the table so she can listen to the lungs. She struggles initially to grab it, but quickly figures it out. She then moves the stethoscope to the virtual patient but struggles to use it in the VR environment. Meanwhile, communication between the nurse and team lead stops while the team lead is task-burdened with learning how to perform an assessment of the lungs in the VR environment. [P4]
Completing interventions in VR	The team lead asks the nurse to give the patient a fluid bolus. The nurse moves to follow the order and realizes that she does not know how to perform the action in the VR environment. The facilitator then proceeds to walk her through the process. [N10]
Fidelity Breakers	
Navigational challenges	
Unintended teleportation	The team lead puts the headset on but finds himself in the ceiling of the virtual environment. He attempts to correct but ends up in the floor, spending several moments trying to get in the correct position. [P13]
Trouble walking	The team lead accidentally teleports to a different location in the room, but is able to quickly get back to her original location without assistance. [N9]
Interaction challenges	
Applying interventions to patient	The team has accidentally taken off the mask while attempting a sternal rub. The entire team is discussing how to get the mask back on and struggle to get it appropriately positioned. The facilitator interrupts, acknowledges the attempt to place the mask, and instructs the team to move on as if the mask has been applied. [P11]
Triggering animations	The team is struggling to get the capillary refill animation to trigger. At first the nurse attempts and is unable, then the team lead then joins. Together, they spend 140 seconds trying to trigger the capillary refill animation in several locations—the foot, the hand, and the sternum. Meanwhile the virtual patient has globally poor perfusion, low blood pressure, and tachycardia, which is not addressed because the team is stuck on trying to trigger the animation. [P8, N8]

a VR: virtual reality.

Theme 1, Source of Truth, outlines the hierarchy of informational sources for participants analyzing clinical scenarios, including subthemes related to initial reliance on virtual cues and dynamic reliance based on responses. Theme 2*,* Cognitive Focus, describes the interplay between thinking, communicating, and doing, with subthemes of dynamic task burden based upon scenario complexity and the cognitive demands of the virtual environment. Theme 3, Fidelity Breakers, explores moments where the boundary between real life and simulation becomes most pronounced, leading participants to deviate from their typical behavior in real-life encounters. Subthemes within this theme relate to navigational and interactional challenges.

### Theme 1: Source of Truth

Participants face novel challenges in a VR medical simulation that they may not have encountered in previous traditional simulation experiences. They are immersed in a completely virtual environment, with real-world objects replaced with digital replicas. This immersion requires participants to reorient themselves to the virtual room and their ability to move and interact within it. We gained insights into how participants navigate this new environment and identify reliable sources of information to guide their actions within the simulated medical case.

#### Subtheme 1.1: Initial Reliance on Virtual Cues

The VR environment offers a visually realistic virtual patient within a replicated clinical environment. This immersive environment replaces the real world with a digital replica, requiring participants to acclimate briefly before focusing on dynamic elements within the room. As participants acclimated to the virtual environment, their focus aligned to three major sources of information: (1) the virtual manikin, (2) the cardiorespiratory monitor, and (3) other participants in the same simulation.

After orientation to the VR environment, participants consistently prioritized soliciting data from the virtual patient. This early prioritization was evident in their frequent comments on the patient’s movements, mental status, and perfusion. The recorded first-person perspective showed that participants spent the majority of their time looking at the patient.

#### Subtheme 1.2: Dynamic Reliance Based on Response

Though initially focused on the virtual patient, participants did use the cardiorespiratory monitor as a secondary source of information. They checked the monitor periodically for brief durations; however, this behavior changed when the patient’s response to interventions deviated from expectations. For example, if the virtual patient’s perfusion did not improve after administration of a fluid bolus, participants shifted to a heavier reliance on the monitor readings, potentially discounting some qualitative physical examination cues in favor of objective data. In such cases, participants became more reliant on the monitor readings, devoting greater attention for extended periods. This shift suggested a transition from using the monitor for confirmation to using it as a primary source of information guiding decision-making.

When both the patient’s presentation and the monitor readings deviated from expected responses to interventions (eg, no immediate improvement in perfusion or heart rate after an intravenous fluid bolus), participants would collaborate to review the case. When working together, participants made decisions based on a combination of group consensus, individual opinions, and real-life clinical algorithms applicable to the context of the simulation (ie, treatment of pneumonia or sepsis). Nonverbal communication was limited due to simulation constraints. Only the participants’ virtual hands and glasses (corresponding to their headset orientation) were visible in the simulation, and thus participants could not assess eye contact or body language as markers of consensus or concern, which is a major deviation from real-life interactions.

If no alternative explanation existed for the perceived incongruity, the participant might seek clarification from the facilitator as the final source of objective data, ranging from asking for clarification on the VR simulation’s functionality to attempting to draw the facilitator into the clinical scenario as an additional participant.

### Theme 2: Cognitive Focus

Participant attention is finite, and the dynamic interplay between the VR environment and participants’ cognitive load unfolded during the simulated scenario. By introducing an entirely virtual world and placing participants in it, VR introduces unique features that may impact the cognitive load and task burden placed upon the participant [30,31]. We observed how the virtual environment affected participants’ ability to process information, make decisions, and execute actions within the simulation. These effects occurred both as an intentional part of the design and as unintended consequences of the VR simulation’s technical limitations.

#### Subtheme 2.1: Task Burden and Scenario Complexity Dynamically Influenced Cognitive Demands

In the initial phase of the simulation, participants faced a high cognitive load as they synthesized the clinical situation while simultaneously familiarizing themselves with the complexities of the VR environment. Participants needed to integrate information from multiple sources: the virtual patient’s visual presentation, physiological data displayed on the monitor, and communication with other team members. As noted in Theme 1, participants initially focused heavily on the virtual patient and the data it provided to anchor their navigation of the clinical scenario.

As the scenario progressed, a critical shift in cognitive demands occurred. The focus transitioned from understanding the patient’s condition to taking decisive actions. Participants needed to execute physical assessments, such as auscultation and capillary refill checks, alongside tasks that included applying supplemental oxygen and initiating intravenous fluid resuscitation. This transition, in many cases, aligns with the natural progression of patient care, moving from initial assessment to formulating and implementing a treatment plan.

#### Subtheme 2.2: Cognitive Demands as an Unexpected Consequence of the Virtual Environment

Cognitive demands as an unintended consequence of the virtual environment (ie, additional demands due to unique VR functionality that differs from real life) impacted participant experience at times. When virtual tasks differed substantially from their real-world counterparts in terms of complexity or intuitiveness, participants appeared to experience increased task burden. We inferred this increased task burden primarily through observed behaviors, such as repeated attempts to perform a task, expressions of frustration, or prolonged fixation on a single action. Certain actions, such as administering a fluid bolus, were intentionally simplified for VR programming feasibility, as it was seen as outside the stated objectives of the training exercise. However, this simplification created a discrepancy from real-world procedures. Experienced nurses, accustomed to a specific bolus administration technique, appeared frustrated by the VR version. This frustration led to disproportionate task fixation on what would otherwise have been a rudimentary task for the team, resulting in excessive focus on technical skills that may have impeded uptake of the simulation’s core learning objectives.

### Theme 3: Fidelity Breakers

Factors intrinsic to the VR environment appeared to challenge participant immersion and disrupt their sense of “being there.” These disruptions, termed Fidelity Breakers, appeared to break participants’ engagement with the simulation and shift their focus from clinical reasoning to technical troubleshooting. The identified Fidelity Breakers fell into the 2 main categories of navigational challenges and interactional challenges.

#### Subtheme 3.1: Navigational Challenges and Learner Adaptations—Limitations in Movement Within the Virtual Space

Participants began each session positioned at the foot of the virtual patient’s bed. Given their starting positioning relative to the patient and the equipment, participants universally tried to move around the room to complete a physical examination or use equipment. However, limitations in the VR system at times disrupted these efforts. Several instances of unintended teleportation were observed: rather than smoothly walking to their desired location via the hand-held controller, participants would inadvertently press a button that transported them to an unintended spot—sometimes on the opposite side of the bed or even partially on top of the patient (refer to Table 1, Fidelity Breakers). Such abrupt relocations caused noticeable confusion and delays while participants struggled to reposition themselves.

As a workaround, several participants elected to lean or reach instead of fully reteleporting to examine the patient more closely. Although this approach facilitated some aspect of examination, it also compromised realism by limiting participants’ freedom to perform typical bedside maneuvers—such as placing a stethoscope on the chest or palpating a pulse—which would otherwise be straightforward during an actual patient encounter. Conversely, some participants became completely fixated on these limitations and spent an outsized amount of time just trying to move around the room, rather than participating in the simulation.

#### Subtheme 3.2: Interaction Challenges—Difficulties Interacting With the Virtual Patient or Equipment

Interactions with the virtual patient and equipment at times appeared to challenge the authenticity of the simulation. Some key examination maneuvers were difficult for participants to perform in VR. For instance, some participants experienced collision detection problems when interacting with the patient. An example includes attempts to apply a supplemental oxygen mask to the virtual patient, which would sometimes accidentally pass through the face (clipping) or fail to trigger the animation altogether. This deviation from real-world actions hindered the intervention and assessment process. Reliance on VR controllers for interactions, as opposed to one’s hands, additionally presented participants with a learning curve that occasionally led to repeated difficulties performing core physical examination skills. A prime example was the challenge of triggering the capillary refill animation, a vital step in evaluating the patient’s hemodynamic status. In some cases, participants became fixated on manipulating the controllers to achieve this specific action, neglecting other aspects of the simulation for several minutes.

## Discussion

### Principal Findings

This study identified 3 major themes regarding the impact of a headset-based VR simulation on participant attention and behavior. VR was found to influence participants to rely primarily on the virtual patient for clinical data and decision-making; however, the participants’ reliance on “Theme 1: Source of Truth” shifted to the cardiorespiratory monitor when the expected patient response to interventions did not occur. In addition, learner behaviors in the VR environment closely reflected the natural progression of patient care, moving from initial assessment to formulating and implementing a treatment plan, allowing closer approximation of the “Theme 2: Cognitive Focus” experienced during clinical care. Finally, participants faced navigational and procedural challenges that were “Theme 3: Fidelity Breakers,” skewing participant attention to the simulation’s technical details rather than the intended learning objectives.

### Participant Reliance on Patient-Centered Data

Practicing medicine in a completely virtual environment is an unfamiliar experience for most medical staff and can be disorienting. In this constructed environment, participants primarily relied on the patient examination as their source of objective data, with focus shifting to the cardiorespiratory monitor only when the virtual patient did not respond as expected to interventions. This grounding of the patient as the center of the simulation, as opposed to a primary reliance on ancillary data, represents a key potential benefit of VR training—reinforcing the patient examination as central in the practice of medicine. Although these observations suggest that VR might help draw learners’ attention to direct patient cues, this conclusion is tentative without a direct comparison to other simulation modalities. While VR could reinforce the patient examination as central in the practice of medicine, further research should verify whether this pattern is unique to VR or equally present in other simulation approaches.

### Limitations of the VR Environment for Procedural Training

Though participants focused on the patient examination, this examination was at times limited. Due to the unique learning curves and inherent technological limitations for movement within the environment and physical manipulation of the patient and equipment, participants often relied primarily on audiovisual cues. In addition, technological glitches causing accidental teleporting and clipping resulted in participants electing to limit their movement around the room, favoring leaning and reaching over moving around the environment, as highlighted through the examples listed in Table 1, Fidelity Breakers. These challenges and resultant learner adaptations suggest that VR might have limitations for learning objectives focused on examination maneuvers or procedures requiring “hands-on” skill development. Until there is no need for technical workarounds or deviations from reality to perform skills in VR, as observed in this study, experience with these tasks in VR may not support skill acquisition that can be directly applied to real-life clinical care. In addition, the cognitive load added by navigating these workarounds may distract from or prevent the acquisition of other learning objectives. Alternatively, these skills may be better learned, practiced, or assessed with alternative modes of simulation, such as computerized manikin-based or AR-enhanced simulation, and thus specific studies examining specific skill acquisition in different modes of simulation are needed.

### Task Fidelity and Cognitive Load Considerations

A virtual patient has the advantage of being able to portray a broad and realistic range of physical findings, as well as dynamic responses to therapy. However, inherent to VR is the requirement of creating digital content for the participant’s entire field of view and expected actions—extending to the environment, equipment, and the interactions with said equipment. Though education and design teams can meticulously craft a virtual environment, any deviations from the real-world environment risk distracting the participant from the primary objectives of the training exercise. This dissonance extends from observable deviations from the real world to divergences in how to perform tasks. Although this VR simulation was designed to focus on recognizing and treating shock, participants were at times distracted by activities outside of that direct focus, such as needing to relearn how to administer a fluid bolus in VR. This intervention was intentionally simplified to reduce the technical learning curve of the simulation, but this simplification had the unintended effect of distracting the learners from the primary focus of the simulation. Future work might explore balancing necessary simplifications with realistic task fidelity to minimize extraneous cognitive load in VR.

### Interface-Specific Training Requirements

VR-based simulation demands additional orientation and training that differ from what is typically provided in manikin-based scenarios. Because certain actions—such as administering a fluid bolus in this scenario (refer to Table 1, N10)—are not replicated 1:1 from real-life procedures, participants must allocate some cognitive load to learning the VR mechanics. This interface-based burden can divert attention from the core clinical objectives, underscoring the importance of structured, VR-specific training sessions before learners engage in complex clinical tasks, and considering these extraneous loads when targeting specific learning objectives.

### Communication and Team Dynamics

Decision-making when treating critical illnesses must be quick to be effective. In VR simulations, team leads made decisions based on a combination of group consensus, individual opinions, and real-life clinical algorithms. Group discussions were frequent, and team communication dynamics appeared to significantly impact decision-making. The VR scenarios included only two participants (plus a facilitator), making decision-making conversational. However, nonverbal communication was limited for this VR simulation, as only participants’ virtual hands and glasses were visualized, which limited assessment of body language and eliminated the ability for eye contact. For this specific VR simulation, the modality encouraged verbal communication around shared observations and findings but lacked feasible options for nonverbal communication. While this reliance on explicit verbal communication may facilitate clarity in certain respects, we cannot determine whether it is more frequent or effective than in traditional simulations without further comparative research. Finally, despite the presence of ongoing communication, individual parallel assessments continued, suggesting that participants processed information independently. A more complex environment or scenario, with potentially a larger team of clinicians and staff, might result in different communication dynamics than what we observed. Assessing the attention and behavior of larger teams in more complex clinical contexts represents key future work for our team.

### Limitations

While our study sheds light on important aspects of VR training for pediatric resuscitation, several limitations warrant consideration. First, the data originated from a single institution over a limited timeframe, encompassing only 15 simulations. While this sample size is relatively small, the participants represent a significant portion of the pediatric resuscitation team at a major academic medical center, potentially fostering generalizability to similar institutions. In addition, data saturation was achieved after analyzing 15 scenarios, indicating that further analyses would not likely yield novel findings. Second, the focused ethnography approach involves inductive analysis, meaning the researchers’ experience and expertise are inherently woven into the methodology. This strengthens the analysis by adding depth and richness to the conclusions drawn. Notably, the research team was purposefully assembled to leverage their expertise in simulation and resuscitation, allowing them to inject diverse perspectives into data analysis and enrich the interpretation of findings.

However, this methodological approach lacks a direct comparator or control group. Because our objective was to describe behaviors in a previously unexamined context, fully immersive VR, we did not measure change from a standard baseline (eg, manikin-based or live simulations). This limitation was partially mitigated by providing rich, context-specific observations from multiple angles, coupled with cross-disciplinary analyst triangulation. Future studies may build on these findings by including mixed method comparisons across different simulation platforms to quantify how VR might augment or hinder specific learning objectives. In addition, there is currently no literature that describes the focus of interdisciplinary teams during real-life clinical care—a key future direction for our team to enhance our ability to align participant behaviors during simulation with those expected during real life.

Finally, this study used a single VR scenario, and thus the conclusions drawn must be considered in relation to the specific technical design and functionality of that scenario. However, while there are a range of VR systems and approaches at various stages of development and availability, the foundational principles of navigating and interacting in a virtual world are likely similar across platforms. As VR technology advances, limitations in movement within the virtual space may be resolved. For example, wireless head-mounted displays allow for easier movement in the VR space by untethering users from stationary computers. Yet even at its best, VR can only approximate—rather than fully replicate—the fidelity of genuine hands-on, kinesthetic practice. Further research can help clarify how these core elements influence participants’ clinical reasoning, teamwork, and skill acquisition, enabling educators to better tailor VR-based simulations to specific learning objectives.

### Conclusions

The conclusions drawn from this study provide a comprehensive understanding of how a VR-based simulation may influence participant attention and behavior, revealing both strengths and limitations of this training modality. Our findings indicate that VR simulations create a unique environment where participants predominantly rely on virtual patient assessments and audiovisual cues. This setup prioritizes patient-centered data over ancillary information, fostering focused clinical decision-making. However, the constraints of VR, such as limited physical interaction and navigational challenges, can impact the authenticity of the training experience, suggesting that VR may be better suited for clinical assessment or cognitive objectives over physical skill–based objectives.

As the landscape of SBME evolves, it is crucial to recognize the potential and limitations of VR simulations compared to other modalities. The ability to create a fully immersive, yet controlled, environment offers significant training advantages for objectives that focus on rapid decision-making and patient assessment. Nevertheless, the technological constraints that limit physical interactions highlight the persistent need for complementary simulation modalities, such as AR and computerized manikin-based simulations, which may better support hands-on skill development. Next steps include the delineation of how the growing armamentarium of simulation modalities and tools can be optimally leveraged to best train a broad range of clinical skills.

## Abbreviations

AR: augmented reality
SBME: simulation-based medical education
VR: virtual reality
